# Immune Responses Following a Fractional Intradermal Second Dose of TAK-003 in Dengue-Seropositive Adults: A Randomized Pilot Trial

**DOI:** 10.3390/vaccines14100887

**Published:** 2026-10-10

**Authors:** Tawin Khaimook, Ratchanon Sophonmanee, Pisud Siripaitoon, Narongdet Kositpantawong, Siripen Kanchanasuwan, Sorawit Chittrakarn, Supattra Uppanisakorn, Nawamin Pinpathomrat, Sarunyou Chusri

**Affiliations:** 1 Infectious Disease Unit, Department of Internal Medicine, Faculty of Medicine, Prince of Songkla University, Songkhla 90110, Thailand; tawan.kh@psu.ac.th (T.K.); grippen45@gmail.com (P.S.); narongdet032@gmail.com (N.K.); kaymed29@yahoo.com (S.K.); sorawit.c@psu.ac.th (S.C.); 2 Department of Biomedical Sciences and Biomedical Engineering, Faculty of Medicine, Prince of Songkla University, Songkhla 90110, Thailand; 6910330021@psu.ac.th (R.S.); nawamin.p@psu.ac.th (N.P.); 3 Clinical Research Center, Faculty of Medicine, Prince of Songkla University, Songkhla 90110, Thailand; poohsupattra@gmail.com

**Keywords:** dengue vaccine, intradermal vaccination, dose-sparing

## Abstract

Background/Objectives: Fractional intradermal administration may offer a dose-sparing approach to dengue vaccination. This randomized pilot study explored the administration of a fractional intradermal second dose of TAK-003 and described immune responses and adverse events in dengue-seropositive adults. Methods: Both groups received a standard 0.5-mL subcutaneous first dose, followed approximately 90 days later by either a standard 0.5-mL subcutaneous second dose or a fractional 0.1-mL intradermal second dose. Twenty-four dengue-seropositive adults were randomized in a 1:1 ratio to the SC–SC or SC–ID group. Anti-dengue IgG and IFN-γ ELISpot responses to DENV peptide pools were assessed at baseline, day 30, and day 120. Immunogenicity analyses were exploratory. Results: All 24 vaccinated participants completed follow-up. Day-30 measurements followed the common subcutaneous first dose and preceded the different second-dose regimens. At day 120, median anti-dengue IgG levels were 141.30 and 146.60 RU/mL in the SC–SC and SC–ID groups, respectively. IFN-γ responses to the tested peptide pools were measurable in both groups. No statistically significant between-group differences were detected at day 120, although the small sample size precludes conclusions of similarity. No serious adverse events were observed. Conclusions: Fractional intradermal administration of the second TAK-003 dose elicited measurable humoral and cellular immune responses and supports the biological feasibility of this dose-sparing approach. Given the exploratory nature and small sample size of this pilot study, these findings warrant confirmation in larger studies.

## 1. Introduction

Dengue fever, caused by the dengue virus (DENV), represents a significant global health challenge, particularly in tropical and subtropical regions. This disease affects millions of individuals annually, resulting in severe morbidity and a substantial economic burden. Advanced age is a significant risk factor for increased severity and mortality associated with this condition [1], predicting an escalating impact in the future. In the absence of specific antiviral treatments, the development and implementation of effective vaccination strategies are crucial in mitigating the impact of dengue fever.

TAK-003 (Qdenga^®^), a live attenuated tetravalent dengue vaccine based on a dengue virus serotype 2 (DENV-2) backbone, has demonstrated efficacy against virologically confirmed dengue and hospitalized dengue in a phase 3 trial [2]. Unlike earlier dengue vaccines, its use does not require prior serological screening, facilitating broader implementation. Nevertheless, improving vaccine accessibility and availability remains an important goal, particularly in dengue-endemic regions.

Several strategies have been proposed to address this challenge. One approach is the development of next-generation vaccines with simplified dosing schedules. The single-dose Butantan Dengue Vaccine (Butantan-DV), derived from the NIH TV003 platform, represents an important advancement in dengue prevention. In a phase 3 evaluation in Brazil, Butantan-DV demonstrated 79.6% efficacy against symptomatic virologically confirmed dengue at 2 years of follow-up, including protection in both seropositive and seronegative individuals [3]. Although a single-dose regimen offers clear logistical advantages and may facilitate vaccine implementation, Butantan-DV is not yet widely available, whereas TAK-003 is currently the most accessible dengue vaccine in many settings, including Thailand.

An alternative strategy to improve vaccine availability is dose sparing through intradermal (ID) administration. The high density of antigen-presenting cells in the dermis provides a biological rationale for investigating immune responses to reduced vaccine doses administered intradermally [4]. A 2009 review commissioned by PATH summarized evidence that reduced intradermal doses could produce immune responses comparable to standard vaccination for some vaccines, particularly influenza and rabies, while findings for other vaccines were mixed or limited [5]. More recent studies have investigated fractional intradermal administration of COVID-19 vaccines [6,7,8]. In resource-limited settings, fractional intradermal dosing may offer a means of extending vaccine supply, although its immunogenicity and reactogenicity require evaluation for each vaccine and regimen.

Continued improvements in dengue vaccination strategies are particularly relevant in Thailand, where dengue seroprevalence exceeds 80% among adults aged ≥20 years and approaches 100% in those aged ≥40 years [9]. The high dengue seroprevalence among Thai adults provides a relevant setting for exploring responses in dengue-experienced individuals, but findings in this population cannot be extrapolated to seronegative individuals.

This randomized pilot trial explored the administration of a fractional 0.1-mL intradermal second dose of TAK-003 following a standard subcutaneous first dose in dengue-seropositive adults. Exploratory immune measurements comprised anti-dengue IgG and IFN-γ ELISpot responses to DENV peptide pools. Day-30 measurements characterized responses following the common first dose, whereas day-120 measurements provided an exploratory comparison after the different second-dose regimens. Adverse events were recorded after each vaccination. The study was not designed to establish equivalence, non-inferiority, protective immunity, or comparative safety.

## 2. Materials and Methods

### 2.1. Study Design and Setting

This was a prospective, single-center, open-label, parallel-group randomized pilot trial with a 1:1 allocation ratio. The study was conducted at the Clinical Research Center, Faculty of Medicine, Prince of Songkla University, Songkhla, Thailand. The trial used an exploratory pilot design and was not designed as a superiority, equivalence, or non-inferiority trial. Participants were recruited in April 2025, and follow-up was completed in September 2025. Participants and members of the public were not involved in the design, conduct, analysis, interpretation, or reporting of this pilot trial. The trial was registered at ClinicalTrials.gov (NCT06837116; first posted on 20 February 2025; https://clinicaltrials.gov/study/NCT06837116 (accessed on 10 September 2026)).

The primary outcome domains specified in the study proposal were anti-dengue antibody levels measured by ELISA and dengue antigen-stimulated responses measured by ELISpot at baseline and days 30 and 120 after the first vaccination. The secondary outcome was the incidence of adverse reactions within 30 days after each vaccination. No single primary assessment time point or between-regimen contrast was designated.

### 2.2. Participants and Eligibility Criteria

Enrollment was restricted to Thai adults aged 18–60 years who were healthy, tested seropositive for anti-dengue IgG at baseline, and had never received a dengue vaccine. Eligibility also required the ability to give written informed consent and the willingness and ability to comply with all study procedures and complete the scheduled follow-up visits through day 120. Screening excluded volunteers with an acute infectious or febrile illness, pregnancy or breastfeeding, or chronic disease that was uncontrolled or clinically significant. Other medical exclusions were a personal or family history of convulsions, epilepsy, encephalopathy, or psychosis; a previous severe allergic reaction to a vaccine or a TAK-003 component; and a clinically significant coagulation disorder or thrombocytopenia. Receipt of immunosuppressive drugs, cytotoxic therapy, or systemic corticosteroids also precluded enrollment, as did receipt of blood products during the 6 months preceding vaccination. Ethical approval was granted on 24 February 2025 by the Human Research Ethics Committee of the Faculty of Medicine, Prince of Songkla University (REC.67-475-14-1). Written informed consent was obtained from every participant before screening and enrollment.

### 2.3. Study Procedures

All participants provided written informed consent before enrollment. Eligible participants were enrolled in this prospective, open-label pilot study and randomly assigned in a 1:1 ratio to the SC–SC or SC–ID group. Before participant enrollment, the random allocation sequence was generated by S. Uppanisakorn, a Clinical Research Center staff member, using Randomizer.org with permuted blocks of four and a 1:1 allocation ratio. The allocation sequence was maintained in sequentially numbered, opaque, sealed envelopes by S. Uppanisakorn. After each participant had been enrolled, the next envelope was opened in sequence to determine the assigned vaccination regimen. Participants and vaccination personnel were not blinded because the administration routes and injected volumes differed. The protocol did not specify a formal blinding or unblinding procedure for laboratory personnel or data analysts.

TAK-003 (QDENGA^®^, Takeda; manufactured by IDT Biologika GmbH, Dessau-Rosslau, Germany; batch no. 562924) was reconstituted with the manufacturer-supplied diluent (0.22% sodium chloride solution) according to the package leaflet. Both subcutaneous and intradermal injections were administered by trained research nurses under investigator supervision using sterile 25-gauge, 16-mm needles supplied with the vaccine packs. The needle specifications were identical for both routes, whereas the injection technique and administered volume differed: 0.5 mL for subcutaneous administration and 0.1 mL for the intradermal second dose. Intradermal injections were performed in the deltoid region, with visible wheal formation used as a procedural indication of intradermal placement. Blood samples were collected immediately before the first vaccination (day 0) and on days 30 and 120. All participants received the second vaccination on day 91; the final blood collection therefore occurred 29 days after the second dose. No predefined feasibility success threshold was specified, and formal implementation outcomes were not systematically assessed. Samples were used for the measurement of anti-dengue IgG levels and IFN-γ ELISpot responses to DENV CD4- and CD8-targeted peptide pools.

### 2.4. Adverse Event Assessment

Immediate adverse events were assessed during a 30-min observation period after each vaccination. Trained research nurses subsequently conducted structured telephone interviews at 7 and 30 days after each dose to ascertain adverse events occurring since vaccination, including events that had resolved before the interview. Participants did not maintain written symptom records; adverse-event ascertainment therefore relied on participant recall. Events were categorized as local or systemic and graded according to treatment requirements: grade 1, no medication required; grade 2, medication required; and grade 3, medical evaluation required.

Seriousness was assessed separately from severity. Serious adverse events were defined as any untoward medical occurrence resulting in death, a life-threatening condition, inpatient hospitalization or prolongation of existing hospitalization, persistent or significant disability or incapacity, or a congenital anomaly/birth defect, regardless of its relationship to the study vaccine. A requirement for medical evaluation alone did not classify an event as serious.

### 2.5. PBMC Isolation

Peripheral blood mononuclear cells (PBMCs) were isolated using density gradient centrifugation. First, 10 mL of whole blood was diluted 1:1 with RPMI 1640 medium and gently layered onto an equal volume of Lymphoprep™ (STEMCELL Technologies, Vancouver, BC, Canada). After centrifugation at 840× *g* for 30 min at 22 °C without a brake, the PBMC layer was collected and washed with RPMI 1640 (30 mL, 840× *g*, 10 min, 22 °C). The pellet was resuspended in 3 mL of RBC lysis buffer for 3 min, washed again with 20 mL of RPMI 1640 (300× *g*, 10 min, 22 °C), and resuspended in 3 mL of RPMI 1640. Cells were counted using 0.4% trypan blue, then washed once more (20 mL, 470× *g*, 5 min, 22 °C). Finally, the pellet was resuspended in RPMI 1640 at a concentration of 5 × 10^6^ cells/mL for assessment of antigen-specific human IFN-γ responses using an ELISpot assay on the day of blood collection.

### 2.6. Serum Collection

Whole blood samples were collected into clot blood tubes and centrifuged at 840× *g* for 10 min at 22 °C to separate the serum. Following centrifugation, the clear serum from the upper phase was carefully transferred into sterile 2 mL screw-cap tubes and stored at −80 °C until further analysis.

### 2.7. ELISA Anti-Dengue IgG

Anti-dengue virus immunoglobulin G (IgG) levels were measured using a commercial ELISA kit (Anti-Dengue Virus ELISA IgG; EUROIMMUN, Lübeck, Germany; product code ERI-EI266b-9601G; lot E251021BO, expiration date 20 January 2027) with a EUROIMMUN Analyzer I-2P (EUROIMMUN) according to the manufacturer’s instructions. Serum samples were diluted 1:4 in sample buffer, and antibody concentrations were derived using the manufacturer-provided calibrators and reported as RU/mL. Aliquots of calibrators, controls, and diluted serum samples (100 µL each) were added to the assay plate and incubated at 37 °C for 60 min. The plates were washed three times with 450 µL of wash buffer per well. Subsequently, 100 µL of peroxidase-labelled anti-human IgG conjugate was added to each well, and the plates were incubated for 30 min at room temperature (18–25 °C). Following washing, 100 µL of chromogen/substrate solution was added to each well, and the plates were incubated for 15 min at room temperature. The reaction was stopped by adding 100 µL of stop solution to each well. Absorbance was measured at 450 nm, with a reference wavelength of 620–650 nm. Results were interpreted according to the manufacturer’s criteria as negative (<16 RU/mL), borderline (≥16 to <22 RU/mL), or positive (≥22 RU/mL). Longitudinal samples from the same participant were tested in the same assay run and on the same plate to minimize inter-assay variability.

### 2.8. ELISpot Anti-Human IFN-γ

DENV-specific cellular responses were assessed using an IFN-γ ELISpot assay (ELISpot Flex: Human IFN-gamma (ALP), Mabtech, Nacka Strand, Sweden; Cat. No. 3420-2A). Freshly isolated PBMCs were used to assess antigen-specific human IFN-γ responses, and PBMC samples with viability ≥80% were considered acceptable for analysis. MultiScreen-IP plates (Millipore, Burlington, MA, USA) were coated overnight at 4 °C with 10 µg/mL anti-human IFN-γ capture antibody (clone 1-D1K, Mabtech) in a carbonate buffer (Sigma-Aldrich, Burlington, MA, USA). Plates were washed with 1× PBS and blocked with R10 medium (RPMI 1640 supplemented with 10% FBS, 1% penicillin/streptomycin and 1% HEPES) for 1 h at 37 °C.

PBMCs were added at 2.5 × 10^5^ cells in 50 µL per well and stimulated in duplicate with 50 µL of either the DENV CD4-targeted (90 peptides; Cat. No. 3698-1) or CD8-targeted (109 peptides; Cat. No. 3695-1) peptide pools at a final concentration of 2 µg/mL of each peptide. Phytohemagglutinin (PHA; Roche Diagnostics GmbH, Mannheim, Germany, Lot. 88811700) at 10 µg/mL was used as a positive control, while R10 medium served as the negative control for background subtraction. Plates were incubated for 16–18 h at 37 °C with 5% CO_2_.

Following incubation, the plates were washed and developed using biotinylated anti-IFN-γ detection antibody (clone 7-B6-1-Biotin, Mabtech), SA-ALP and BCIP/NBT-plus substrate (Mabtech; Cat. No. 3650-10). Spots were counted using an ImmunoSpot Micro-Analyzer (Cellular Technology Limited, Shaker Heights, OH, USA). Samples were tested in duplicate, and the mean number of spots from replicate wells was used for analysis. Background responses from the corresponding unstimulated control wells were subtracted from peptide-stimulated responses and expressed as SFCs/10^6^ PBMCs. Background-subtracted values below zero were assigned a value of zero.

### 2.9. Statistical Analysis

The target sample size of 12 participants per group was selected using the pragmatic pilot-study guidance proposed by Julious [10]; no formal power calculation for between-group immunogenicity or safety comparisons was performed. Continuous outcomes were summarized using medians and interquartile ranges. Between-group comparisons used two-sided Mann–Whitney U tests. Mann–Whitney U-test *p* values were calculated using the asymptotic method with correction for tied ranks. Day-120 between-regimen effects were summarized using Hodges–Lehmann location-shift estimates (SC–ID minus SC–SC) with 95% confidence intervals. Within-group comparisons across the three visits used Friedman tests followed by Dunn’s adjustment for pairwise comparisons. No overall multiplicity adjustment was applied across immune endpoints and between-group comparisons. All immunogenicity analyses were exploratory, and *p*-values were interpreted alongside the observed distributions and the limitations of the sample size. For adverse-event outcomes, proportions are presented with 95% exact Clopper–Pearson confidence intervals. Between-group absolute risk differences (SC–ID minus SC–SC) are presented with 95% confidence intervals calculated using the Newcombe method based on Wilson score intervals. These methods were selected because of the small sample size and sparse event counts, including zero-event cells. Analyses were performed using GraphPad Prism version 10.6.1 (GraphPad Software, LLC, Boston, MA, USA). No interim analyses were planned or performed. Prespecified termination criteria included conflict with governmental policies, new scientific evidence indicating no benefit from study continuation, or severe local reactions attributable to intradermal vaccination in more than 10% of participants. Post hoc descriptive summaries were prepared by vaccination regimen and age stratum (≤37 and >37 years), using the overall median age as the cut-off. Given the small total sample and sparse age-by-regimen subgroups, no hypothesis tests comparing age strata or age-adjusted regression analyses were performed.

## 3. Results

### 3.1. Study Participants

This study was conducted at the Clinical Research Center, Faculty of Medicine, Prince of Songkla University, Songkhla, Thailand. Demographics of the study participants are shown in Table 1. Table 2 summarizes humoral and cellular immune responses at each study visit, within-group longitudinal comparisons, and day-120 between-group effect estimates. All randomized participants completed the scheduled follow-up visits. No outcome data were missing for the analyses reported, and no imputation was performed.

**Figure 1:**
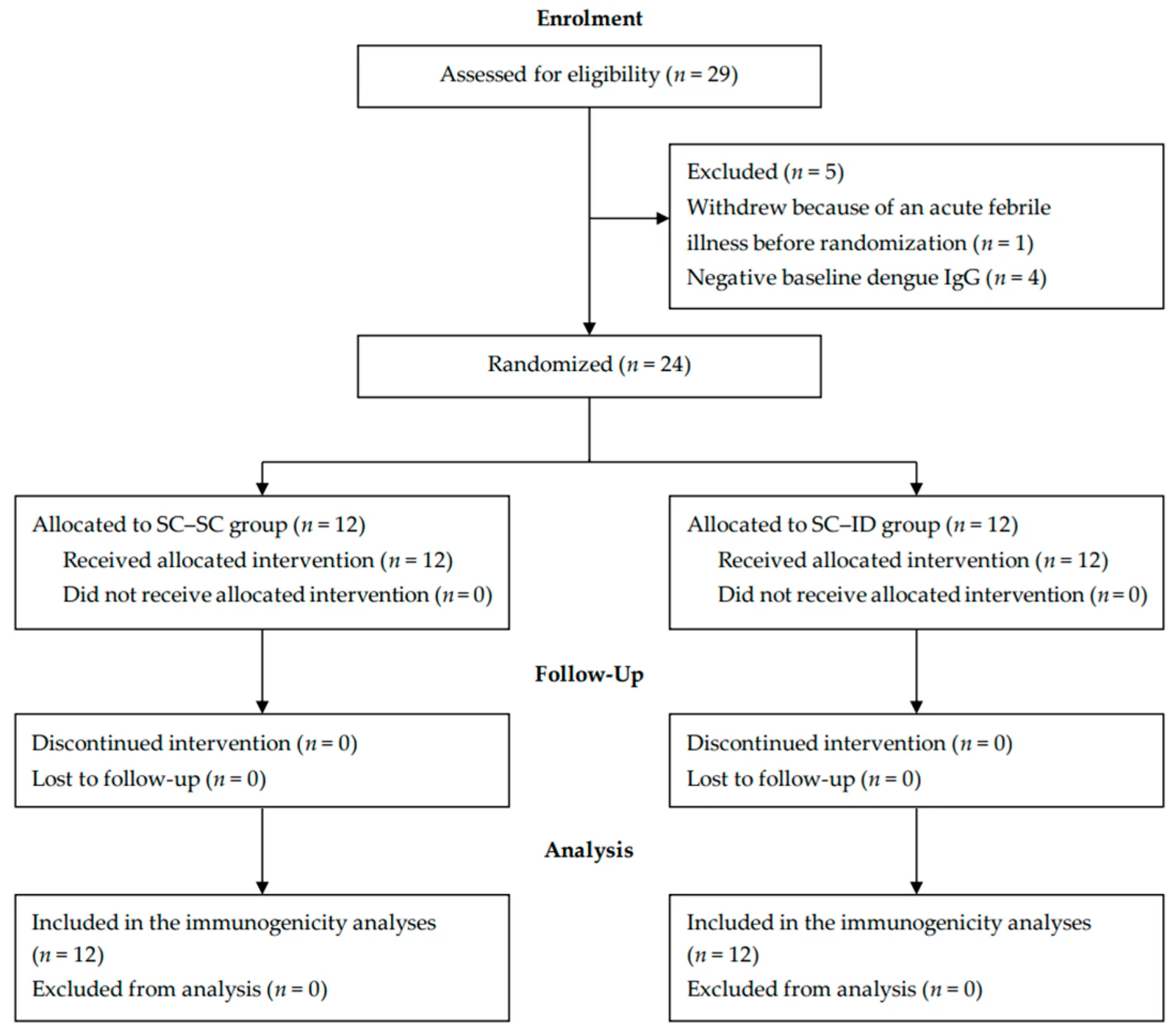
CONSORT diagram.

Twenty-nine volunteers were assessed for eligibility. Five were excluded before randomization: four had negative baseline anti-dengue IgG results, and one withdrew because of an acute febrile illness before randomization. Twenty-four participants were randomly assigned to the SC–SC group (*n* = 12) or the SC–ID group (*n* = 12). All 12 participants allocated to the SC–ID group received the planned 0.1-mL intradermal second dose, administered by trained research nurses under investigator supervision. All randomized participants received the allocated intervention, completed the scheduled follow-up, and were included in the analyses. No participant was lost to follow-up or excluded after randomization (Figure 1).

### 3.2. Immunogenicity

Both groups received the same subcutaneous first dose at day 0. Day-30 measurements were obtained before the second vaccination. Day-120 measurements were obtained approximately 30 days after the different second-dose regimens. Results are presented separately for these two assessment periods within each immune outcome.

#### 3.2.1. Anti-Dengue IgG Responses

Responses following the initial subcutaneous dose

**Figure 2:**
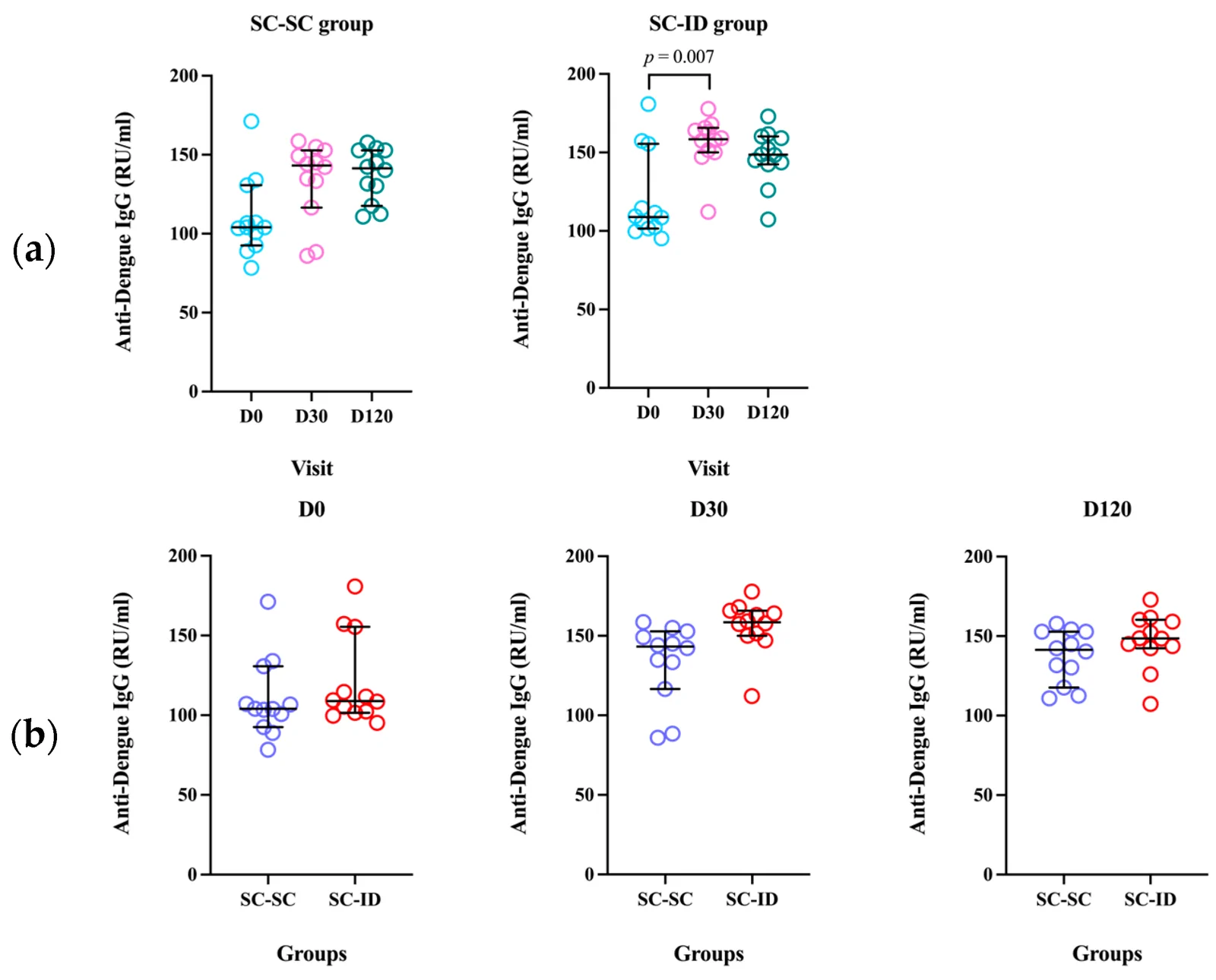
Antibody (anti-dengue IgG) responses to vaccination. (**a**) Anti-dengue IgG responses within-group comparison at D0, D30, and D120. (**b**) Anti-dengue IgG responses between-group comparison at each visit. Data are presented as medians with IQR (*n* = 12 per group). Within-group comparisons were performed using the Friedman test followed by Dunn’s multiple-comparison test, and between-group comparisons were performed using the Mann–Whitney U test. D0 denotes baseline before the common subcutaneous first dose; D30 denotes 30 days after the first dose and before the second vaccination; and D120 denotes approximately 30 days after the standard subcutaneous or fractional intradermal second dose.

From baseline to day 30, median anti-dengue IgG levels increased from 104.00 (IQR, 94.59–124.80) to 143.20 (120.80–151.90) RU/mL in the SC–SC group (Dunn-adjusted *p* = 0.307), and from 108.90 (101.70–145.20) to 158.50 (150.40–165.30) RU/mL in the SC–ID group (Dunn-adjusted *p* = 0.007). At day 30, before either group received the second dose, IgG levels were higher in the SC–ID group than in the SC–SC group (Mann–Whitney U *p* = 0.003; Table 2; Figure 2).

Exploratory comparison following the second-dose intervention

At day 120, median anti-dengue IgG levels were 141.30 (IQR, 120.80–152.80) RU/mL in the SC–SC group and 146.60 (142.70–160.00) RU/mL in the SC–ID group. No statistically significant between-group difference in anti-dengue IgG levels was detected at day 120 (Mann–Whitney U *p* = 0.160; Figure 2b). Within-group comparisons of day 120 with baseline and day 30 are presented in Table 2.

#### 3.2.2. IFN-γ ELISpot Responses

Responses following the initial subcutaneous dose

**Figure 3:**
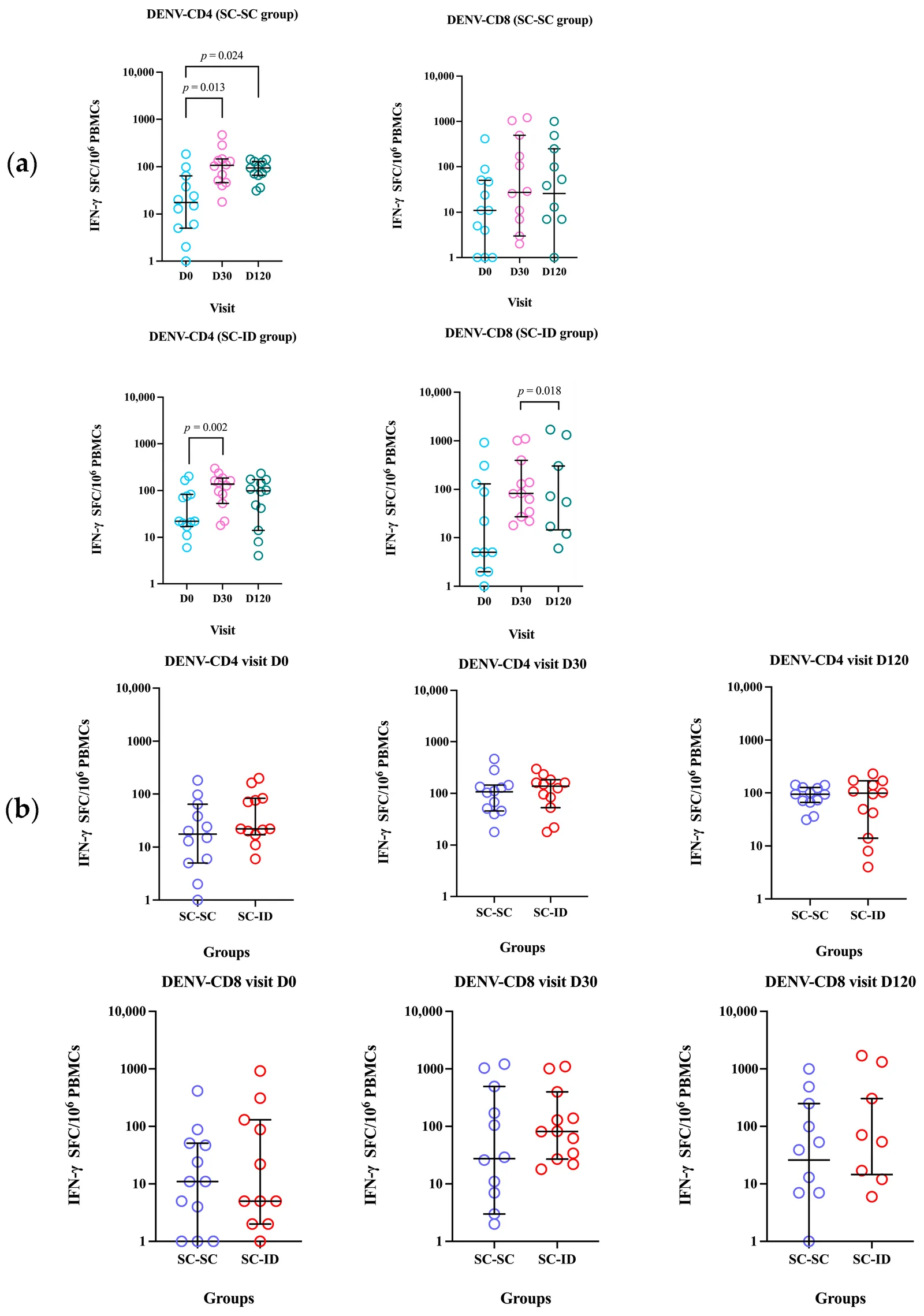
IFN-γ ELISpot responses to DENV peptide pools following vaccination. (**a**) IFN-γ ELISpot responses to CD4- and CD8-targeted DENV peptide pools in the SC–SC and SC–ID groups at D0, D30, and D120. (**b**) IFN-γ ELISpot responses to CD4- and CD8-targeted DENV peptide pools for the two vaccination regimens (SC–SC vs. SC–ID). Data are presented as medians with IQR (*n* = 12 per group). Within-group comparisons across time points were performed using the Friedman test followed by Dunn’s multiple-comparison test, and between-group comparisons were performed using the Mann–Whitney U test. D0 denotes baseline before the common subcutaneous first dose; D30 denotes 30 days after the first dose and before the second vaccination; and D120 denotes approximately 30 days after the standard subcutaneous or fractional intradermal second dose.

IFN-γ ELISpot responses were measured following stimulation with DENV CD4- and CD8-targeted peptide pools and expressed as spot-forming cells (SFCs) per 10^6^ PBMCs. From baseline to day 30, median responses to the CD4-targeted pool increased from 17.50 (IQR, 5.25–57.50) to 107.00 (47.25–142.30) in the SC–SC group (Dunn-adjusted *p* = 0.013), and from 22.00 (17.75–81.25) to 137.50 (60.75–178.30) in the SC–ID group (Dunn-adjusted *p* = 0.002). Median responses to the CD8-targeted pool increased numerically from 11.00 (1.75–50.00) to 27.50 (4.00–413.00) in the SC–SC group (Dunn-adjusted *p* = 0.057), and from 5.00 (2.00–119.50) to 81.50 (28.75–332.50) in the SC–ID group (Dunn-adjusted *p* = 0.096). Between-group comparisons at day 30 yielded *p* = 0.523 for the CD4-targeted pool and *p* = 0.319 for the CD8-targeted pool (Mann–Whitney U tests; Table 2; Figure 3).

Exploratory comparison following the second-dose intervention

At day 120, median responses to the CD4-targeted pool were 94.50 (IQR, 67.25–127.00) and 98.50 (21.00–163.80) SFCs/10^6^ PBMCs in the SC–SC and SC–ID groups, respectively (Mann–Whitney U *p* = 0.966). Corresponding responses to the CD8-targeted pool were 26.00 (2.50–211.50) and 14.50 (0.00–245.80) SFCs/10^6^ PBMCs (Mann–Whitney U *p* = 0.831; Figure 3b). Within-group comparisons of day 120 with baseline and day 30 are presented in Table 2. Neither the CD4-targeted nor the CD8-targeted peptide-pool response differed significantly between regimens at day 120.

Overall, no statistically significant differences between the SC–SC and SC–ID regimens were detected at day 120 in either anti-dengue IgG levels or IFN-γ responses to the CD4- and CD8-targeted peptide pools. These exploratory findings do not establish equivalence between regimens.

Participant-level longitudinal changes in humoral and cellular immune responses are shown in Supplementary Figure S1. Post hoc age-stratified immunogenicity summaries are presented in Table S4. The SC–SC group included four participants aged ≤37 years and eight aged >37 years, compared with eight and four, respectively, in the SC–ID group. At day 120, median anti-dengue IgG levels in the SC–SC versus SC–ID groups were 148.27 versus 148.57 RU/mL among participants aged ≤37 years and 136.02 versus 153.33 RU/mL among those aged >37 years. Median IFN-γ responses to the CD4-targeted pool were 135.0 versus 78.0 and 72.0 versus 98.5 SFCs/10^6^ PBMCs in the younger and older strata, respectively; corresponding responses to the CD8-targeted pool were 369.0 versus 14.5 and 10.0 versus 30.0 SFCs/10^6^ PBMCs.

### 3.3. Immediate and Delayed Adverse Events

No immediate adverse events were observed during the 30-min observation period after either vaccination. All participants received the first dose subcutaneously, whereas the second dose was administered subcutaneously or intradermally according to group assignment. During the study, six participants (three per group) experienced at least one AE, corresponding to 25.0% in each group.

Across both vaccination visits, local reactions were reported in 25.0% of participants in the SC–ID group and 8.3% in the SC–SC group (risk difference = +16.7 percentage points, 95% CI −14.8 to 45.7). The most common local reactions were injection-site pain (8.3% SC–SC vs. 16.7% SC–ID) and redness (0% SC–SC vs. 25.0% SC–ID). Systemic events (malaise and headache) were reported only in the SC–SC group at the day-7 follow-up after the first vaccination.

After the second vaccination, no adverse events were reported in the SC–SC group (0/12; 0.0%, 95% CI 0.0–26.5%), whereas two participants in the SC–ID group reported adverse events (2/12; 16.7%, 95% CI 2.1–48.4%), both consisting of local redness. The absolute risk difference was +16.7 percentage points (95% CI −10.4 to 44.8). No systemic adverse events were reported after the second dose.

All recorded adverse events were grade 1 and required no medication. No serious adverse events were identified during the scheduled assessments through day 30 after either dose. No grade 3 adverse events or deaths occurred. Exact symptom onset, duration, and resolution, and adverse events between day 30 and the second vaccination, were not systematically documented. A summary of all adverse events is provided in Table S1. Adverse events reported after the first and second vaccine doses are presented separately in Tables S2 and S3, respectively.

## 4. Discussion

Previous phase 1 research evaluated subcutaneous and intradermal administration of a live attenuated tetravalent dengue vaccine candidate in flavivirus-naive adults [11]. In the present study, we adopted a stepwise exploratory design in which all participants received the standard subcutaneous first dose before evaluation of a fractional intradermal second dose. This design assessed a mixed-route two-dose schedule rather than an entirely intradermal regimen. Because both dose and route differed at the second vaccination, their individual contributions could not be separated.

This randomized pilot study described immune measurements following a fractional intradermal second dose of TAK-003 in dengue-seropositive adults who had received a standard subcutaneous first dose. Day-120 measurements were the only observations obtained after the groups received different second-dose regimens. Although no statistically significant between-group differences were detected at that visit, the small sample size does not establish similarity, equivalence, or non-inferiority. The day-30 IgG difference occurred before intradermal administration and therefore cannot support an effect of the intradermal second dose. Fractional intradermal administration was performed in the study setting, but broader implementation feasibility was not formally assessed. This observation was limited to delivery of the planned dose; predefined technical success criteria and formal implementation outcomes were not assessed. It therefore does not establish feasibility for routine vaccination programs.

Our research group previously evaluated the immunogenicity and reactogenicity of fractional intradermal COVID-19 vaccine boosters [12,13], but those findings cannot establish the immunogenicity or safety of intradermal TAK-003. In the present study, six of 24 vaccinated participants reported at least one adverse event, and no serious adverse events were observed. The cumulative local-reaction estimate was imprecise and included events after both vaccination visits. Events following the second dose are reported separately (Table S3). These observations characterize adverse events in this small cohort and do not establish comparative safety or exclude rare serious adverse events.

An important consideration when interpreting our findings is that all participants were dengue-seropositive adults residing in an endemic setting. In Thailand, dengue seroprevalence among adults exceeds 80% [9], indicating that most individuals have been previously exposed to one or more dengue virus serotypes. Consequently, vaccine implementation in endemic populations differs fundamentally from vaccination of dengue-naïve travelers, as pre-existing immunity may substantially influence vaccine-induced responses. Seropositive individuals already possess dengue-specific memory B-cells and T-cells that can be rapidly reactivated upon antigen re-exposure. In this context, intradermal administration may function primarily as a booster of established immune memory rather than as a priming immunization. Pre-existing immune memory may have contributed to the measured responses, but this mechanism was not directly established by the assays used. The findings apply to dengue-seropositive adults and cannot be generalized to seronegative individuals, children, or an entirely intradermal vaccination schedule.

Notably, TAK-003 has demonstrated sustained protection and immune persistence well beyond the first year. Overall vaccine efficacy remained 61% against virologically confirmed dengue and 84% against hospitalized dengue through approximately 4.5 years of follow-up [2]. Long-term T-cell responses following TAK-003 vaccination have been reported [14]. Results from NCT03999996 include serotype-specific neutralizing antibody geometric mean titers and seropositivity rates through Month 42 [15]. These findings support the durability of the DENV-2 backbone platform [16]. Published long-term findings for standard TAK-003 vaccination provide context but cannot be extrapolated to the fractional intradermal second-dose regimen studied here. Our last immune assessment was approximately 30 days after the second vaccination, which does not establish the durability of responses following that regimen.

This pilot study has some limitations, including the small sample size, short follow-up period, and lack of neutralizing antibody measurements or memory T-cell phenotyping. Similarly, IFN-γ responses to pooled DENV peptides did not resolve responses to individual serotypes and therefore could not establish the breadth or balance of cellular immunity across the four dengue serotypes. A substantial age imbalance remained despite randomization: the median age was 42.5 years in the SC–SC group and 28.5 years in the SC–ID group. Randomization does not guarantee covariate balance in a small trial. Age may be associated with prior dengue or other flavivirus exposure and with immune responsiveness, although individual exposure histories were not fully characterized. This imbalance is an important source of uncertainty when interpreting the observed between-group responses. Post hoc age-stratified summaries were provided to describe the observed distributions. However, the small numbers within each age-by-regimen subgroup limit interpretation, and these descriptive summaries do not establish an age effect or account for the age imbalance in the between-regimen comparison. The humoral assessment was limited to anti-dengue IgG measured by ELISA. Serotype-specific neutralizing antibody responses were not assessed, and the results therefore do not establish functional antibody activity, tetravalent breadth, or protection. Baseline anti-dengue IgG positivity was not independently confirmed and may have been influenced by cross-reactive antibodies. Histories of other flavivirus infections and vaccinations were not systematically recorded. Although no fever was reported during scheduled post-vaccination assessments, intercurrent dengue infection was not investigated by laboratory testing. Its contribution to subsequent immune measurements therefore cannot be excluded or quantified. This limitation is particularly important in a population with pre-existing dengue immunity. These factors limit the ability to draw definitive conclusions regarding long-term protection and immune correlates of efficacy. As a pilot study, the trial was not powered to establish equivalence or non-inferiority between the second-dose regimens. Additional adequately powered longitudinal studies with extended follow-up, including neutralizing antibody assays and memory T-cell analyses, are needed to better define the durability of immune responses and their relationship to long-term protection.

## 5. Conclusions

Fractional intradermal administration of the second TAK-003 dose elicited measurable humoral and cellular immune responses, with no serious adverse events observed in this small pilot study. These findings support the biological feasibility of intradermal boosting as a potential dose-sparing approach but require confirmation in larger studies with longer follow-up.

## Figures and Tables

**Table 1:** Baseline characteristics of the study participants.

Parameter	Overall (*n* = 24)	SC–SC (*n* = 12)	SC–ID (*n* = 12)
Age, y (median [IQR])	37.0 (27.8–43.0)	42.5 (35.0–43.0)	28.5 (26.0–38.8)
Sex: n (%) Female	21 (87.5)	11 (91.7)	10 (83.3)
Pre-vaccination systolic blood pressure, mmHg, median (IQR)	120.0 (110.0–131.8)	119.5 (110.8–126.5)	122.0 (107.0–134.3)
Pre-vaccination diastolic blood pressure, mmHg, median (IQR)	76.0 (69.3–86.3)	78.5 (69.3–90.3)	76.0 (68.5–81.3)

**Table 2:** Summary of humoral and cellular immune responses.

Parameter	SC–SC (*n* = 12)	SC–ID (*n* = 12)	Between-Group Comparison
Humoral response: anti-dengue IgG (RU/mL)			
Baseline	104.00 (94.59–124.80)	108.90 (101.70–145.20)	*p* = 0.291
Day 30	143.20 (120.80–151.90)	158.50 (150.40–165.30)	*p* = 0.003
Day 120	141.30 (120.80–152.80)	146.60 (142.70–160.00)	*p* = 0.160
Dunn-adjusted within-group comparisons			
Day 30 vs. baseline	*p* = 0.307	*p* = 0.007	—
Day 120 vs. day 30	*p* > 0.999	*p* = 0.662	—
Day 120 vs. baseline	*p* = 0.459	*p* = 0.199	—
Day-120 between-group effect	+8.62 (95% CI −4.36 to 27.30); *p* = 0.160		
IFN-γ response to the CD4-targeted peptide pool (SFCs/10^6^ PBMCs)			
Baseline	17.50 (5.25–57.50)	22.00 (17.75–81.25)	*p* = 0.213
Day 30	107.00 (47.25–142.30)	137.50 (60.75–178.30)	*p* = 0.523
Day 120	94.50 (67.25–127.00)	98.50 (21.00–163.80)	*p* = 0.966
Dunn-adjusted within-group comparisons			
Day 30 vs. baseline	*p* = 0.013	*p* = 0.002	—
Day 120 vs. day 30	*p* > 0.999	*p* = 0.124	—
Day 120 vs. baseline	*p* = 0.024	*p* = 0.459	—
Day-120 between-group effect	+0.50 (95% CI −57.00 to 64.00); *p* = 0.966		
IFN-γ response to the CD8-targeted peptide pool (SFCs/10^6^ PBMCs)			
Baseline	11.00 (1.75–50.00)	5.00 (2.00–119.50)	*p* = 0.988
Day 30	27.50 (4.00–413.00)	81.50 (28.75–332.50)	*p* = 0.319
Day 120	26.00 (2.50–211.50)	14.50 (0.00–245.80)	*p* = 0.831
Dunn-adjusted within-group comparisons			
Day 30 vs. baseline	*p* = 0.057	*p* = 0.096	—
Day 120 vs. day 30	*p* = 0.307	*p* = 0.018	—
Day 120 vs. baseline	*p* > 0.999	*p* > 0.999	—
Day-120 between-group effect	−0.50 (95% CI −53.00 to 64.00); *p* = 0.831		

Data are presented as median (interquartile range) unless otherwise indicated. Between-group comparisons at each visit were performed using two-sided Mann–Whitney U tests. Within-group comparisons across visits were performed using Friedman tests followed by Dunn-adjusted pairwise comparisons. Day-120 between-group effects are expressed as Hodges–Lehmann location-shift estimates (SC–ID minus SC–SC) with 95% confidence intervals. CI, confidence interval; DENV, dengue virus; PBMCs, peripheral blood mononuclear cells; RU, relative units; SFCs, spot-forming cells.

## Data Availability

The individual-level data generated in this study are not publicly available because the participant consent did not include public data deposition. De-identified data and the study protocol may be made available by the corresponding author upon reasonable request, subject to approval by the relevant ethics committee and an appropriate data-sharing agreement. No separate statistical analysis plan was prepared.

## Supplementary Materials

The following supporting information can be downloaded at: https://www.mdpi.com/article/10.3390/vaccines14100887/s1, Figure S1, Individual longitudinal humoral and cellular immune responses following vaccination; Table S1, Total adverse events across both vaccine doses; Table S2, Adverse events after the first vaccine dose; Table S3, Adverse events after the second vaccine dose; Table S4, Age-stratified humoral and cellular immunogenicity responses.

## Clinical Trial Registration

This study was prospectively registered at ClinicalTrials.gov (NCT06837116; first posted on 20 February 2025; https://clinicaltrials.gov/study/NCT06837116 (accessed on 10 September 2026)).

## Abbreviations

The following abbreviations are used in this manuscript:

AE	adverse event
DENV	dengue virus
ELISA	enzyme-linked immunosorbent assay
ID	intradermal
IFN-γ	interferon gamma
IQR	interquartile range
PBMC	peripheral blood mononuclear cell
SC	subcutaneous
SFC	spot-forming cell
TAK-003	live attenuated tetravalent dengue vaccine
WHO	World Health Organization

## References

[1] Lin R.J., Lee T.H., Leo Y.S. (2017). Dengue in the elderly: A review. Expert Rev. Anti-Infect. Ther..

[2] Tricou V., Yu D., Reynales H., Biswal S., Saez-Llorens X., Sirivichayakul C., Lopez P., Borja-Tabora C., Bravo L., Kosalaraksa P. (2024). Long-term efficacy and safety of a tetravalent dengue vaccine (TAK-003): 4·5-year results from a phase 3, randomised, double-blind, placebo-controlled trial. Lancet Glob. Health.

[3] Kallás E.G., Cintra M.A.T., Moreira J.A., Patiño E.G., Braga P.E., Tenório J.C.V., Infante V., Palacios R., de Lacerda M.V.G., Batista Pereira D. (2024). Live, Attenuated, Tetravalent Butantan-Dengue Vaccine in Children and Adults. N. Engl. J. Med..

[4] Schaumburg F., De Pijper C.A., Grobusch M.P. (2019). Intradermal travel vaccinations—When less means more. Travel Med. Infect. Dis..

[5] Hickling J., Jones R. (2009). Intradermal Delivery of Vaccines: A Review of the Literature and the Potential for Development for Use in Low- and Middle-Income Countries.

[6] Roozen G.V.T., Granger A., van Binnendijk R.S., den Hartog G., Roestenberg M., Visser L.G., Roukens A.H.E. (2024). Intradermal fractional dose vaccination as a method to vaccinate individuals with suspected allergy to mRNA COVID-19 vaccines. Vaccine.

[7] Roozen G.V.T., Prins M.L.M., van Binnendijk R., den Hartog G., Kuiper V.P., Prins C., Janse J.J., Kruithof A.C., Feltkamp M.C.W., Kuijer M. (2022). Safety and Immunogenicity of Intradermal Fractional Dose Administration of the mRNA-1273 Vaccine: A Proof-of-Concept Study. Ann. Intern. Med..

[8] Prins M.L.M., Roozen G.V.T., Pothast C.R., Huisman W., van Binnendijk R., den Hartog G., Kuiper V.P., Prins C., Janse J.J., Lamers O.A.C. (2024). Immunogenicity and reactogenicity of intradermal mRNA-1273 SARS-CoV-2 vaccination: A non- inferiority, randomized-controlled trial. npj Vaccines.

[9] Sonthichai C., Manadee T., Yoocharoen P. (2023). Seroprevalence of Dengue virus infection among Thai population. Dis. Control J..

[10] Julious S.A. (2005). Sample size of 12 per group rule of thumb for a pilot study. Pharm. Stat..

[11] George S.L., Wong M.A., Dube T.J., Boroughs K.L., Stovall J.L., Luy B.E., Haller A.A., Osorio J.E., Eggemeyer L.M., Irby-Moore S. (2015). Safety and Immunogenicity of a Live Attenuated Tetravalent Dengue Vaccine Candidate in Flavivirus-Naive Adults: A Randomized, Double-Blinded Phase 1 Clinical Trial. J. Infect. Dis..

[12] Intapiboon P., Seepathomnarong P., Ongarj J., Surasombatpattana S., Uppanisakorn S., Mahasirimongkol S., Sawaengdee W., Phumiamorn S., Sapsutthipas S., Sangsupawanich P. (2021). Immunogenicity and Safety of an Intradermal BNT162b2 mRNA Vaccine Booster after Two Doses of Inactivated SARS-CoV-2 Vaccine in Healthy Population. Vaccines.

[13] Pinpathomrat N., Intapiboon P., Seepathomnarong P., Ongarj J., Sophonmanee R., Hengprakop J., Surasombatpattana S., Uppanisakorn S., Mahasirimongkol S., Sawaengdee W. (2022). Immunogenicity and safety of an intradermal ChAdOx1 nCoV-19 boost in a healthy population. npj Vaccines.

[14] Mandaric S., Friberg H., Saez-Llorens X., Borja-Tabora C., Biswal S., Escudero I., Faccin A., Gottardo R., Brose M., Roubinis N. (2024). Long term T cell response and safety of a tetravalent dengue vaccine in healthy children. npj Vaccines.

[15] ClinicalTrials.gov A Phase 3, Follow-Up Trial to Evaluate Long-Term Safety and Antibody Persistence, and the Impact of a Booster Dose of a Tetravalent Dengue Vaccine Candidate in Healthy Adolescents and Adults in Areas Non-Endemic for Dengue (NCT03999996). Results. NCT03999996.

[16] Sáez-Llorens X., DeAntonio R., Low J.G.H., Kosalaraksa P., Dean H., Sharma M., Tricou V., Biswal S. (2025). TAK-003: Development of a tetravalent dengue vaccine. Expert Rev. Vaccines.

