# Supplementary material for: Immune Responses Following a Fractional Intradermal Second Dose of TAK-003 in Dengue-Seropositive Adults: A Randomized Pilot Trial
Source: Vaccines (Basel). 2026 Oct 10;14(10):887. doi: 10.3390/vaccines14100887 (PMC13709505; doi:10.3390/vaccines14100887)
Supplement: Supplementary file 1 [file vaccines-14-00887-s001.zip › Figure S1. Individual longitudinal humoral and cellular immune responses following vaccination.pdf]

(a) (b) (c)

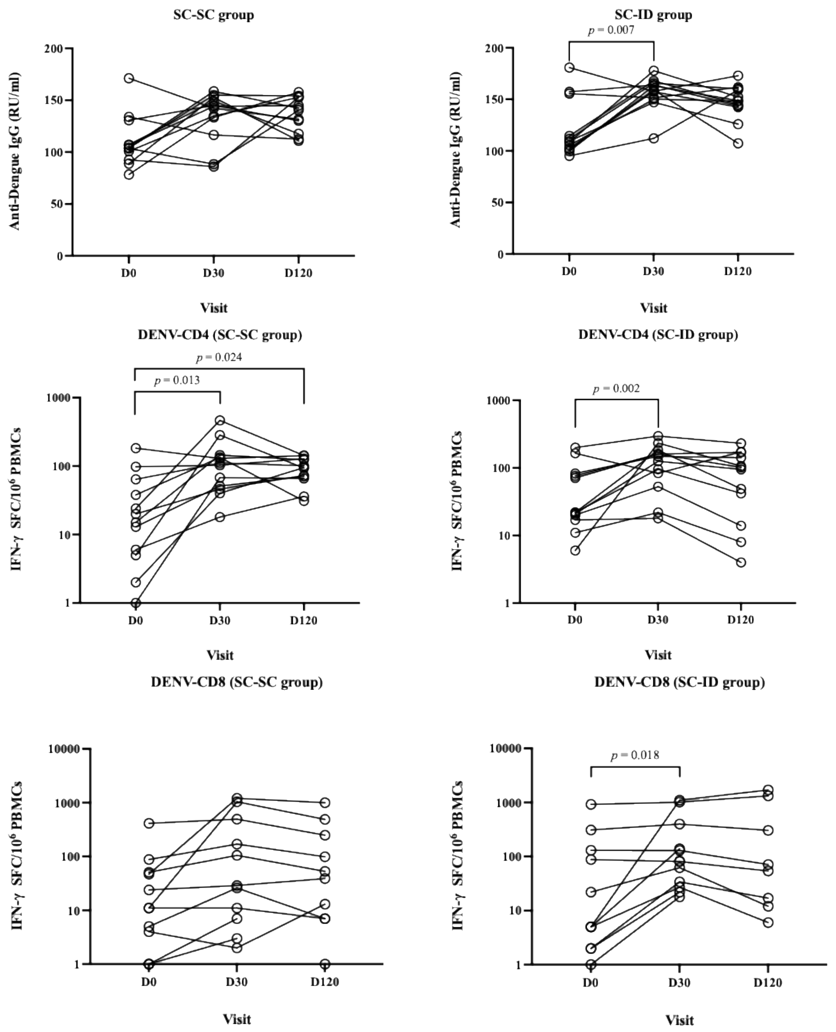

Figure S1. Individual longitudinal humoral and cellular immune responses following vaccination
(a) Individual longitudinal anti-dengue IgG responses at D0, D30, and D120.
(b) Individual longitudinal IFN-γ ELISpot responses to the CD4-targeted DENV peptide pool at D0, D30, and D120.
(c) Individual longitudinal IFN-γ ELISpot responses to the CD8-targeted DENV peptide pool at D0, D30, and D120.
Each line represents an individual participant, with measurements connected across D0, D30, and D120. Individual trajectories are shown separately for the SC–SC and SC–ID regimens.
