# Supplementary material for: Immune Responses Following a Fractional Intradermal Second Dose of TAK-003 in Dengue-Seropositive Adults: A Randomized Pilot Trial
Source: Vaccines (Basel). 2026 Oct 10;14(10):887. doi: 10.3390/vaccines14100887 (PMC13709505; doi:10.3390/vaccines14100887)
Supplement: Supplementary file 1 [file vaccines-14-00887-s001.zip › Table S1-S3 Adverse events with CL 9-10-69.pdf]

## Adverse events

**Table S1. Total adverse events across both vaccine doses**

| Adverse event | SC–SC (*n* = 12), *n*/*N* (%) [95% CI] | SC–ID (*n* = 12), *n*/*N* (%) [95% CI] | Risk difference, SC–ID – SC–SC, % (95% CI) |
|---|---|---|---|
| Any AE | 3/12 (25.0) [5.5–57.2] | 3/12 (25.0) [5.5–57.2] | 0.0 (−32.5 to 32.5) |
| Local reactions | 1/12 (8.3) [0.2–38.5] | 3/12 (25.0) [5.5–57.2] | +16.7 (−14.8 to 45.7) |
| Pain | 1/12 (8.3) [0.2–38.5] | 2/12 (16.7) [2.1–48.4] | +8.3 (−21.3 to 37.3) |
| Redness | 0/12 (0.0) [0.0–26.5] | 3/12 (25.0) [5.5–57.2] | +25.0 (−4.1 to 53.2) |
| Systemic reactions | 2/12 (16.7) [2.1–48.4] | 0/12 (0.0) [0.0–26.5] | −16.7 (−44.8 to 10.4) |
| Malaise | 1/12 (8.3) [0.2–38.5] | 0/12 (0.0) [0.0–26.5] | −8.3 (−35.4 to 16.9) |
| Headache | 1/12 (8.3) [0.2–38.5] | 0/12 (0.0) [0.0–26.5] | −8.3 (−35.4 to 16.9) |
| Serious AEs | 0/12 (0.0) [0.0–26.5] | 0/12 (0.0) [0.0–26.5] | 0.0 (−24.2 to 24.2) |

Data are presented as the number of participants experiencing at least one adverse event, *n*/*N* (%), with 95% confidence intervals calculated using the exact Clopper–Pearson method. Between-group risk differences are expressed as SC–ID minus SC–SC and are presented with 95% confidence intervals calculated using the Newcombe method based on Wilson score intervals. Table S1 summarizes participant-level adverse events occurring after either vaccine dose; participants experiencing the same category of event after both doses were counted once within that category. All recorded adverse events were grade 1 and required no medication. AE, adverse event; CI, confidence interval; SC–SC, subcutaneous first and second doses; SC–ID, subcutaneous first dose followed by fractional intradermal second dose.

**Table S2. Adverse events after the first vaccine dose**

| Adverse event | SC–SC (*n* = 12), *n*/*N* (%) [95% CI] | SC–ID (*n* = 12), n/N (%) [95% CI] | Risk difference, SC–ID – SC–SC, % (95% CI) |
|---|---|---|---|
| Any AE | 3/12 (25.0) [5.5–57.2] | 2/12 (16.7) [2.1–48.4] | −8.3 (−39.0 to 24.1) |
| Local reactions | 1/12 (8.3) [0.2–38.5] | 2/12 (16.7) [2.1–48.4] | +8.3 (−21.3 to 37.3) |
| Pain | 1/12 (8.3) [0.2–38.5] | 2/12 (16.7) [2.1–48.4] | +8.3 (−21.3 to 37.3) |
| Redness | 0/12 (0.0) [0.0–26.5] | 2/12 (16.7) [2.1–48.4] | +16.7 (−10.4 to 44.8) |
| Systemic reactions | 2/12 (16.7) [2.1–48.4] | 0/12 (0.0) [0.0–26.5] | −16.7 (−44.8 to 10.4) |
| Malaise | 1/12 (8.3) [0.2–38.5] | 0/12 (0.0) [0.0–26.5] | −8.3 (−35.4 to 16.9) |
| Headache | 1/12 (8.3) [0.2–38.5] | 0/12 (0.0) [0.0–26.5] | −8.3 (−35.4 to 16.9) |
| Serious AEs | 0/12 (0.0) [0.0–26.5] | 0/12 (0.0) [0.0–26.5] | 0.0 (−24.2 to 24.2) |

Data are presented as *n*/*N* (%) with 95% confidence intervals calculated using the exact Clopper–Pearson method. Between-group risk differences are expressed as SC–ID minus SC–SC and are presented with 95% confidence intervals calculated using the Newcombe method based on Wilson score intervals. Adverse events shown in Table S2 occurred following the first vaccine dose, which was administered subcutaneously in both groups. All recorded adverse events were grade 1 and required no medication. AE, adverse event; CI, confidence interval; SC–SC, subcutaneous first and second doses; SC–ID, subcutaneous first dose followed by fractional intradermal second dose.

**Table S3. Adverse events after the second vaccine dose**

| Adverse event | SC–SC (*n* = 12), *n*/*N* (%) [95% CI] | SC–ID (*n* = 12), *n*/*N* (%) [95% CI] | Risk difference, SC–ID − SC–SC, % (95% CI) |
|---|---|---|---|
| Any AE | 0/12 (0.0) [0.0–26.5] | 2/12 (16.7) [2.1–48.4] | +16.7 (−10.4 to 44.8) |
| Local reactions | 0/12 (0.0) [0.0–26.5] | 2/12 (16.7) [2.1–48.4] | +16.7 (−10.4 to 44.8) |
| Pain | 0/12 (0.0) [0.0–26.5] | 0/12 (0.0) [0.0–26.5] | 0.0 (−24.2 to 24.2) |
| Redness | 0/12 (0.0) [0.0–26.5] | 2/12 (16.7) [2.1–48.4] | +16.7 (−10.4 to 44.8) |
| Systemic reactions | 0/12 (0.0) [0.0–26.5] | 0/12 (0.0) [0.0–26.5] | 0.0 (−24.2 to 24.2) |
| Malaise | 0/12 (0.0) [0.0–26.5] | 0/12 (0.0) [0.0–26.5] | 0.0 (−24.2 to 24.2) |
| Headache | 0/12 (0.0) [0.0–26.5] | 0/12 (0.0) [0.0–26.5] | 0.0 (−24.2 to 24.2) |
| Serious AEs | 0/12 (0.0) [0.0–26.5] | 0/12 (0.0) [0.0–26.5] | 0.0 (−24.2 to 24.2) |

Data are presented as n/N (%) with 95% confidence intervals calculated using the exact Clopper–Pearson method. Between-group risk differences are expressed as SC–ID minus SC–SC and are presented with 95% confidence intervals calculated using the Newcombe method based on Wilson score intervals. Adverse events shown in Table S3 occurred following the second vaccine dose, when the SC–SC group received the standard subcutaneous dose and the SC–ID group received the fractional intradermal dose. All recorded adverse events were grade 1 and required no medication. AE, adverse event; CI, confidence interval; SC–SC, subcutaneous first and second doses; SC–ID, subcutaneous first dose followed by fractional intradermal second dose.
