# Supplementary material for: Immune Responses Following a Fractional Intradermal Second Dose of TAK-003 in Dengue-Seropositive Adults: A Randomized Pilot Trial
Source: Vaccines (Basel). 2026 Oct 10;14(10):887. doi: 10.3390/vaccines14100887 (PMC13709505; doi:10.3390/vaccines14100887)
Supplement: Supplementary file 1 [file vaccines-14-00887-s001.zip › Table S4 Age-stratified humoral and cellular immunogenicity responses 9-10-69.pdf]

Table S4 *Age-stratified humoral and cellular immunogenicity responses.*

Group	SC-SC ≤37 years (n = 4)	SC-SC >37 years (n = 8)	SC-ID ≤37 years (n = 8)	SC-ID >37 years (n = 4)
**Humoral response (Anti-dengue IgG, ELISA, RU/mL)**				
Baseline	104.03 (97.55-104.85)	105.09 (98.76-131.56)	106.97 (101.72-109.96)	135.00 (111.28-161.78)
Day 30	152.10 (145.24-155.90)	138.59 (109.55-144.37)	161.14 (149.31-164.46)	157.63 (155.96-160.35)
Day 120	148.27 (136.17-155.10)	136.02 (125.79-146.85)	148.57 (139.22-153.95)	153.33 (144.34-164.48)
**IFN-γ response to the CD4-targeted peptide pool (SFC per 10^6 PBMCs)**				
Baseline	61.0 (19.25-119.25)	14.0 (5.0-24.5)	21.0 (15.5-74.0)	49.0 (21.75-98.25)
Day 30	206.5 (122.50-329.50)	59.0 (44.50-116.75)	155.0 (42.25-196.50)	111.0 (93.0-134.75)
Day 120	135.0 (119.50-142.25)	72.0 (58.50-96.50)	78.0 (12.50-149.25)	98.5 (81.75-119.75)
**IFN-γ response to the CD8-targeted peptide pool (SFC per 10^6 PBMCs)**				
Baseline	29.0 (11.0-138.75)	4.5 (1.0-30.75)	3.5 (1.75-49.0)	46.5 (5.0-296.5)
Day 30	765.0 (373.25-1078.50)	16.5 (2.75-48.0)	72.0 (31.0-196.0)	110.0 (67.50-357.25)
Day 120	369.0 (188.50-611.25)	10.0 (0.75-42.50)	14.5 (0-129.25)	30.0 (4.50-371.25)

Participants were stratified by the overall median age into those aged ≤37 years and >37 years. Anti-dengue IgG levels (RU/mL) and IFN-γ ELISpot responses to CD4- and CD8-targeted DENV peptide pools (SFCs/10^6 PBMCs) are presented as medians (interquartile ranges) by vaccination regimen and visit. D0 denotes baseline; D30 denotes 30 days after the common subcutaneous first dose and before the second vaccination; and D120 denotes approximately 30 days after the second vaccination. These post hoc summaries are descriptive; no hypothesis tests or age-adjusted regression analyses were performed.
